# Firearm Exposure and Storage Practices in the Homes of Rural Adolescents

**DOI:** 10.5811/westjem.2021.3.50263

**Published:** 2021-05-19

**Authors:** Charles A. Jennissen, Kristel M. Wetjen, Cole C. Wymore, Nicholas R. Stange, Gerene M. Denning, Junlin Liao, Kelly E. Wood

**Affiliations:** * University of Iowa Carver College of Medicine, Department of Emergency Medicine, Iowa City, Iowa; † University of Iowa Carver College of Medicine, Stead Family Department of Pediatrics, Iowa City, Iowa; ‡ University of Iowa Stead Family Children's Hospital, Department of Surgery, Iowa City, Iowa; § University of Iowa Hospitals and Clinics, Department of Surgery, Iowa City, Iowa; ¶ Saint Louis University School of Medicine, St. Louis. Missouri

## Abstract

**Introduction:**

Rural areas have higher rates of firearm-related unintentional and suicide deaths. Having access to a firearm greatly increases suicide risk. Safe firearm storage can be a major factor in preventing these tragedies. In this study we evaluated firearm exposure and storage practices in rural adolescents’ homes.

**Methods:**

An anonymous survey was administered to a convenience sample of attendees at the 2019 Iowa FFA (formerly Future Farmers of America) Leadership Conference. We performed descriptive, bivariate and multivariable logistic regression analyses.

**Results:**

A total of 1,382 adolescents participated; 51% were males and 49% were females. Respondents were 13–18 years old, and 53% lived on a farm, 18% in the country/not on a farm, and 29% in town. Almost all (96%) self-identified as White/Caucasian. In their homes, 84% reported having rifles/shotguns, 58% reported having handguns, and 56% reported having both rifles/shotguns and handguns. Males were significantly more likely than females to report having firearms in their home (P<0.001). The likelihood of having rifles/shotguns was greater if living on a farm (odds ratio (OR) 4.19, 95% confidence interval (CI), 2.99–5.88) or in the country/not a farm (OR 2.74, 95% CI, 1.78–4.24) compared to those in town. Similarly, the presence of handguns in the home was increased if living on a farm compared to in town (OR 1.70, 95% CI 1.32–2.18). Rifles/shotguns and handguns were stored unlocked and/or loaded at least some of the time in 62% and 58% of homes, respectively. Those who lived on farms compared to in towns were more likely to have rifles/shotguns (OR 1.83, 95% CI 1.35–2.46) and handguns (OR 1.58, 95% CI 1.10–2.27) stored unlocked. For homes with unlocked rifles/shotguns, 46% stored ammunition unlocked. For homes with unlocked handguns, 38% stored ammunition unlocked. Among those aware of firearm storage in their home, 82% (802/974) reported at least one firearm stored either unlocked and/or loaded at least some of the time.

**Conclusion:**

The vast majority of rural adolescents we surveyed live in homes with firearms, and a large proportion of those firearms are not stored safely. Widespread efforts are needed to educate rural families about the importance of proper firearm and ammunition storage.

## INTRODUCTION

Firearm-related injuries in the United States (US) are the second leading cause of child and adolescent death, and the nation’s pediatric mortality rate from firearms is the highest in the world.^1^–^3^ According to World Health Organization data, the US pediatric firearm-related unintentional and suicide death rates for victims 5–14 years old were 12 and 11 times greater, respectively, than those of 23 other industrialized countries.^3^ The firearm-related death rate for children 0–4 years old was 33 times higher.^3^ Moreover, from 2013–2017 the fatality rate for youth in the US increased by 44%.^4^

Although pediatric firearm injuries may be intentional (eg. homicide, suicide), a large proportion are unintentional. In fact, more than half of pediatric admissions for firearm injuries in children 15 years and younger are for unintentional injuries.^5^, ^6^ The majority of unintentional firearm fatalities in children occur in the home, and most occur when the child is playing with a loaded firearm.^7^ In the US, approximately one-third of homes with children have a firearm present, and it is estimated that approximately 4.6 million US children live in homes with a firearm stored unlocked and loaded.^8^, ^9^

Suicide rates among America’s youth are increasing, and tripled for those 10–14 years old from 1999 to 2014.^10^–^12^ Suicide attempts by firearms are highly lethal with over 90% resulting in death.^13^ In one study, 65% of youths who committed or attempted suicide by firearm obtained the gun from their home.^14^ Having access to a firearm increases the likelihood of suicide among youth.^11^

Several studies have shown that firearm-related unintentional and suicide death rates are higher in rural as compared to urban US counties.^15^–^17^ In 2019, the Firearm Safety Among Children and Teens (FACTS) Consortium identified as a research priority understanding how the availability, storage, and presence of a firearm in the home affects youth outcomes.^18^ The objective of our study was to determine firearm exposure and storage practices in the homes of rural adolescents, and to identify demographic factors associated with having firearms present and unsafely stored in the home.

## METHODS

### Study Population

This was a cross-sectional survey study of a convenience sample of adolescents attending the 2019 Iowa FFA Leadership Conference. FFA (formerly known as Future Farmers of America) is a national organization with local chapters in all 50 states and Puerto Rico. Membership is free, and the organization offers students leadership, personal growth, and career success training through agricultural education. Conference attendees volunteered and anonymously completed a written survey at the study institution’s injury prevention booth. Surveys were completed independently and reviewed by safety-booth staff for completeness. Following the survey, participants were given the opportunity to ask questions about gun safety, offered printed safety materials, and allowed to spin a wheel for a small prize. All conference attendees were eligible to complete the survey, but study analysis was restricted to those 13–18 years of age.

**Population Health Research Capsule**

What do we already know about this issue?

*Rural areas have higher rates of firearm-related unintentional and suicide deaths. In the majority of these tragedies, the gun involved was obtained from the home.*

What was the research question?

*We sought to determine firearm exposure and storage practices in the homes of rural adolescents who attended a state conference.*

What was the major finding of the study?

*Eighty-five percent of adolescents lived in a home with a firearm. In many homes, firearms and ammunition were stored unsafely.*

How does this improve population health?

*Understanding firearm practices in the homes of rural adolescents will lead to evidence-based education to help prevent firearm-related death and injury.*

### Survey

The survey was developed at the study institution by members of the Injury Prevention Task Force and other individuals interested in firearm injury prevention through a collaborative and iterative process. The survey tool was validated by 20 youth and young adults ages 11–22 years. After completing the written survey, these volunteers explained their responses to the questions and were asked to clarify their answers if a question was not understood. Verbal and written responses to questions were compared for consistency. The survey was revised based on the results.

Demographic data collected included age (years), gender (male, female, other), residence (on a farm, in the country/not on a farm, in town), and race (White/Caucasian, Black/African American, Hispanic Latinx, Asian, other). The five individuals who answered “other” for gender were not included in comparative analyses. Races/ethnicities besides White/Caucasian were categorized as “other races” for study purposes. Study data collected included the presence of firearms and firearm storage methods in the participant’s home. On the survey, the term “firearm” was defined as a weapon “from which a bullet or other projectile is fired by gunpowder,” and did not include BB guns, pellet guns, or dart guns. The term “home” included “the place you sleep and all other buildings your family owns on the same property.” A firearm was considered “unlocked” if it was “not locked in a storage place or not stored with a trigger lock or cable.”

Participants were asked if there were any rifles/shotguns and/or handguns in their home with responses “yes” and “not that I know of.” The latter was used instead of “no” as some adolescents may not be aware of firearms in the home. If the respondent answered “yes” for either the presence of rifles/shotguns or handguns, they were separately asked if these firearms were stored loaded, unlocked, or both loaded and unlocked. Answers for each included “Yes/Always,” “Yes/Sometimes,” “No,” and “Not sure.” Those responding “Not sure” were not included in comparative analyses. A firearm was considered safely stored if it was always stored unloaded and locked. Any firearm stored at least sometimes loaded and/or unlocked was considered unsafely stored.

### Data Analysis

The surveys were completed on paper and provided to the research team for analysis. The institutional review board deemed the research exempt as analysis was done on an anonymously collected existing dataset. We entered data into survey software (Qualtrics International, Inc, Provo, UT). Aggregate results were then exported as an Excel spreadsheet (Microsoft Corp, Redmond, WA) and imported into Stata 15.1 (StataCorp, College Station, TX). We performed descriptive (frequencies), bivariate (chi square, Fisher’s exact test), and multivariable logistic regression analyses. All *P*-values were two-tailed, and a value <0.05 was considered statistically significant. Missing data were not included in analyses.

## RESULTS

### Subject Demographics

A total of 1382 adolescents were included in analysis. The proportion of males and females was nearly equivalent (Table 1). Almost two-thirds were 16–18 years old. More than half lived on a farm, almost one-fifth resided in the country/not on a farm, and 29% lived in town. The vast majority (96%) were White/Caucasian.

### Firearms in the Home

Over four-fifths (84%) of respondents reported that at least one rifle or shotgun was present in their homes, and 58% reported the presence of at least one handgun (Table 1). More than one-half (56%) indicated that both rifles/shotguns and handguns were present in their homes. Only 2% of respondents reported having handguns only, and 27% reported rifles/shotguns only.

### Comparison of Rifle/Shotgun Presence in the Home

Males, older teens, and participants identifying as White/Caucasian had significantly higher percentages reporting a rifle/shotgun in the home as compared to their peers (Table 2). Participants who lived on a farm more frequently reported rifles/shotguns in the home than those residing in the country/not on a farm, (*P*<0.0001). Additionally, a higher proportion of both of these groups reported the presence of rifles/shotguns in their homes as compared to respondents who lived in town. Logistic regression analysis demonstrated that males were 2.4 times more likely than females, and that other races were 40% less likely than Whites/Caucasians to report at least one rifle/shotgun in the home. Those living on a farm and those living in the country/not on a farm were 4.2 and 2.7 times more likely, respectively, to report the presence of rifles/shotguns in the home than those residing in town.

### Comparison of Handgun Presence in the Home

Significantly more male respondents reported handguns in the home as compared to females (Table 3). In terms of residence, the frequency of reporting the presence of a handgun in the home was on a farm > in the country/not on a farm > in town, overall *P*<0.001. Logistic regression analysis showed that males were 1.35 times more likely than females to report having a handgun in the home, and those living on a farm were 1.70 times more likely to report a handgun than those residing in a town. No differences were seen by race/ethnicity.

### Firearm Storage Practices in the Home

Among those aware of rifle/shotgun storage practices in their homes, almost one-third reported they were stored sometimes or always loaded, and over one-half reported they were stored sometimes or always unlocked (Table 4). Almost one-fifth reported the rifles/shotguns were stored both loaded and unlocked at least some of the time. Overall, only one-third of those with rifles/shotguns in their home indicated they were safely stored at all times, ie, always stored unloaded and locked.

As for adolescents aware of handgun storage in their home, two-fifths reported the handguns were stored loaded, nearly one-half reported they were stored unlocked, and over one-fourth reported they were stored both loaded and unlocked at least some of the time. Like rifles/shotguns, only about one-third of youth with handguns in their home reported they were always stored safely (unloaded and locked). Of respondents overall who were aware of how firearms were stored in their homes, over four-fifths (802/974, 82.3%) reported at least one firearm was stored either unlocked or loaded at least some of the time.

### Comparison of Rifle/Shotgun Storage in the Home

As compared to their peers, males, older adolescents, and Whites/Caucasians more frequently reported having at least sometimes unlocked rifles/shotguns in the home (Table 5). When comparing storage by residence location, the percentage reporting unlocked rifles/shotguns in their homes was in the following order: those living on farms > those living in the country/not on a farm > those living in town, overall *P*<0.001. Logistic regression analysis indicated that males were 1.8 times more likely than females, older teenagers were 1.3 times more likely than younger teenagers, and those living on a farm were 1.8 times more likely than those residing in town to report at least one rifle/shotgun always or sometimes stored unlocked in their homes. In contrast to results for unlocked rifles/shotguns, there were no significant demographic differences with respect to rifles/shotguns being stored loaded in the home.

### Comparison of Handgun Storage in the Home

Males had significantly higher percentages reporting handguns were loaded, unlocked, and both loaded and unlocked as compared to females (Table 6). For those living on a farm, a greater percentage also reported unlocked handguns than those living elsewhere, *P*<0.001. Logistic regression analysis showed males were 1.6 times, 1.7 times, and 2.7 times more likely than females to report having handguns stored loaded, unlocked, and both loaded and unlocked, respectively. Those living on farms were 1.6 times more likely than those from towns to report an unlocked handgun at least some of the time.

### Ammunition Storage Practices

Trends were similar when analyzing storage of ammunition for both rifles/shotguns and handguns (Table 7). For those aware of rifle/shotgun ammunition storage in their homes, 28% said the ammunition was stored unlocked, 31% stated it was locked with the firearms, and 41% reported it was stored and locked separately from the firearms, ie, safely. Among those aware of handgun ammunition storage practices, 25% stated it was stored unlocked in the home, 36% that it was locked with the handguns, and 40% that it was stored and locked separately. Although there were a number of differences among variables and ammunition storage in the home, the only consistent finding was that those living on farms as compared to those living in towns were 1.9 and 1.8 times more likely to have unlocked rifle/shotgun and handgun ammunition, respectively. Those reporting firearms were stored at least sometimes unlocked in the home were significantly more likely to also report unlocked ammunition as compared to respondents in homes where firearms were always kept locked, *P*<0.001 for both rifles/shotguns and handguns.

### Presence of Firearms in the Homes Adolescents Visit

Eighty-five percent (1168/1382) of respondents reported visiting homes with firearms. These homes included those of family members (86%), friends (82%), neighbors (47%), and others (11%). Males had higher proportions than females (90%, 629/696 vs 79%, 537/680, *P*<0.001) and Whites/Caucasians had greater percentages than other races (85%, 1124/1320 vs. 70%, 43/61, *P* = 0.002) with respect to having visited homes with firearms. There were no differences by survey participant age or residence location. In logistic regression analysis, males were 2.4 times more likely than females (95% CI, 1.77–3.32) and other races were 60% less likely than Whites/Caucasians (95% CI, 0.24–0.79) to report having visited homes with firearms. The firearms in the homes they visited were similar to the firearms in their own homes overall with 69% of the homes having both rifles/shotguns and handguns, 16% had rifles/shotguns only, and 2% had handguns only. Twelve percent of participants were not sure of the types of firearms present.

## DISCUSSION

We surveyed adolescent FFA members living in a rural state to learn about firearm exposure and storage practices in their homes. In our study, the vast majority of adolescents lived in a home with a firearm, with 84% having at least one rifle/shotgun and over half having handguns. Only 15% in our study had no firearms in their home. In addition, over four-fifths of the adolescents reported visiting homes that contained a firearm. Significant proportions of both rifles/shotguns and handguns in survey respondents’ homes were stored loaded and/or unlocked at least some of the time. In fact, among those aware of storage in their home, more than four-fifths reported having at least one firearm loaded and/or unlocked at least some of the time. Moreover, those with unlocked firearms had significantly greater proportions with unlocked ammunition as compared with homes where firearms were always kept locked.

### Characteristics of Firearms in the Home

The proportion of rural youth in our study who lived in a home with at least one firearm was twice that found in a national 2017 Pew Research Center survey of all Americans.^19^ Similarly, eight Gallup polls from December 2012–October 2019 found that 37–43% of US homes had a firearm.^20^ The higher proportion observed in our study is consistent with numerous studies showing more frequent gun ownership in rural as compared to urban residences.^19^,^21^–^24^ It is also consistent with a study of 983 households in one rural Iowa county from 1994–1998 where two-thirds of residents reported at least one firearm.^25^

Although rifles/shotguns were more common, handguns were present in over one-half of the homes in our study. Several studies of firearm injuries and deaths seen at rural trauma centers have shown handguns to be the most common firearm used and the ones most frequently involved in fatal cases.^26^–^28^ Our findings of rural homes having high handgun ownership is also consistent with other studies that have demonstrated a high prevalence of handgun carrying among rural youth.^29^,^30^

Firearms in the home varied significantly based on where the adolescent lived, with significantly higher rates seen for those living on a farm or in the country but not on a farm. Our results mirror what has been reported in surveys of adults with the highest rates of firearm ownership for those living in rural, followed by suburban, and then urban areas.^19^,^31^ Hunting is likely a major contributor to the higher rates of rifle/shotgun presence in the homes of adolescents living on farms and in the country.^31^ One study found only 2% of metropolitan residents hunted as compared to 18% in cities of <50,000 people.^32^ In many rural areas, hunting is a part of the culture and receiving a rifle or shotgun as an adolescent is a rite of passage.^33^

Males in our study were significantly more likely than females to report having a firearm in their home. The basis for this difference remains unknown. However, other studies have shown that males, especially White/Caucasian males, are more likely to own and/or to have grown up in a home with firearms.^19^,^31^ Additionally, adolescent males in rural areas are more likely to have engaged in recreational firearm use and to have expressed pro-gun sentiments than their female peers.^19^,^34^ Boys are disproportionately affected by firearm mortality accounting for more than 80% of all pediatric firearm deaths.^7^ The higher rate of home firearms we observed with males may potentially be a contributing factor to this gender-based difference.

### Firearm and Ammunition Storage

Unsafe storage of firearms in the homes of rural youth in our study was high. Among those aware of storage practices, the vast majority (82%) reported at least one firearm stored unsafely at least some of the time. A Washington state study reported nearly two-thirds of adult respondents with firearms stated they were not all safely stored (eg, both locked and unloaded).^35^ Additionally, we found higher percentages of handguns, as compared to rifles/shotguns, were reported as being stored loaded and unlocked. Consistent with this observation are studies showing that the primary reason Americans state they have a firearm is for protection, and that firearms kept for protection, handguns in particular, are often stored loaded and unlocked for quick access.^36^–^37^ Unfortunately, unsafe storage practices increase the risk of unintentional and self-inflicted firearm injuries in children and adolescents as is illustrated by studies showing firearms in the home are much more likely to kill or injure a household member than to be used in self-defense.^38^,^39^

### Firearms in Homes Visited

Over four-fifths of adolescents (85%) visited homes, most typically of family members or friends, that contained a firearm. The majority had both rifles/shotguns and handguns. Visiting a home with a firearm can be dangerous especially for younger adolescents. In one study of youth 11–14 years of age, nearly 40% of unintentional firearm deaths happened at the home of a friend, which was a proportion higher than that reported for younger children.^40^ The authors speculated that the difference may be accounted for by decreased adult supervision of adolescents as compared to younger children.^40^

### Societal Implications

The results of our study suggest that rural adolescents in our state are a very vulnerable population. Previous research has shown that firearm-related unintentional and self-inflicted injuries and hospitalizations are higher in rural than in urban areas^15^,^16^,^27^,^41^ Similarly, rural youth are three times more likely to die by suicide as compared to their urban counterparts.^16^,^42^ The greater presence of firearms in rural homes as well as the relatively high prevalence of improper storage likely contribute to the disproportionate rates of rural adolescent firearm-related injuries and suicides.

### Prevention

To protect children and adolescents, parents and caregivers must prevent unwanted access to firearms.^11^,^43^ The safest option would be to remove the firearm from the home, but as seen in our study, rural adolescents have potential access to firearms in the homes of others as well. The second most effective prevention approach is safe storage practices, particularly in homes where youth live and visit. Thus, widespread education and interventional programs are critically needed regarding the safe storage of firearms and ammunition. Another critically important measure is the passage of universal child access prevention (CAP) laws to protect children equally across states and to better ensure the safe storage of firearms in homes.^5^,^44^–^51^ Enforcement of these laws that hold parents and other relevant adults accountable when children and adolescents access firearms in the home might provide a strong impetus for more widespread safe storage of ammunition and firearms.^52^,^53^ Reducing child and adolescent firearm access in turn could decrease unintentional and self-inflicted pediatric firearm-related deaths and injuries.^10^,^54^

## LIMITATIONS

Limitations of our study include that it was conducted in a single Midwestern state with a primarily White/Caucasian population. Thus, our findings may not be generalizable to other states and non-White populations. Additionally, we used a convenience sampling of adolescent FFA members primarily from rural areas attending a state conference; therefore, results may not be representative of the entire state, particularly urban communities. However, the great majority of counties in the state were represented by subjects in the study. Data was self-reported and may be subject to recall bias and social desirability. With regard to social desirability, participants would probably have been more likely to report safe rather than unsafe storage practices. Factors decreasing the social desirability effect included the fact that the surveys were written, completed independently, and collected anonymously.

It is possible that some study participants’ homes had firearms of which the youth were unaware. Thus, the overall proportion of homes with firearms may be higher than that reported. In addition, there were some survey respondents who were unsure of at least one of the three firearm storage questions including 12% (142/1156) of those with rifles/shotguns and 11% (92/801) with handguns. These responses were not included in Table 4 calculations. Similarly, some adolescents were unsure how ammunition was stored in the home (9% for rifle/shotgun and 10% for handguns). Females and younger teenagers had higher proportions unsure of firearm and ammunition storage.

## CONCLUSION

The vast majority of rural adolescents in this study lived in a home with a firearm and many reported firearms and ammunition were stored unsafely. The likelihood of having a firearm in the home varied significantly based upon where the adolescent lived with highest rates for those living on a farm. Rural families would benefit from education about the importance of safe storage of firearms and ammunition to limit unwanted child and adolescent access. Consideration of the unique cultural and social aspects of rural communities is necessary to develop effective injury prevention strategies for this setting. The implementation of strict and well-enforced universal childhood access prevention laws may be a critical step in protecting youth from firearm-related tragedies.

## Figures and Tables

**Table 1:** Demographic and firearm-related variables of adolescent survey respondents.

	n (Col%)^a^
Group N	1382
Gender	
Male	697 (51%)
Female	680 (49%)
Age	
13 years	29 (2%)
14 years	120 (9%)
15 years	330 (24%)
16 years	363 (26%)
17 years	321 (23%)
18 years	219 (16%)
Residence	
Farm	727 (53%)
Country/not a farm	250 (18%)
Town	400 (29%)
Race	
White/Caucasian	1,320 (96%)
Other races	61 (4%)
Rifle/shotgun in home	
Yes	1,159 (84%)
Not that I know of	223 (16%)
Handgun in home	
Yes	802 (58%)
Not that I know of	580 (42%)
Combined firearms in home	
Both rifle and handgun	780 (56%)
Rifle/shotgun only	379 (27%)
Handgun only	22 (2%)
None that I know of	201 (15%)

a The sum of n may not equal the total Group N due to missing values.

**Table 2:** Bivariate and multivariate logistic regression analyses regarding the presence of rifles/shotguns in the homes of adolescent survey respondents.

	Crosstab analysis			Logistic regression analysis	
	
	Yes n (Row %)^b^	No^a^ n (Row %)^b^	P-value	Odds ratio	Confidence interval
Group N	1,159 (84%)	223 (16%)			
Gender			*P* < 0.001		
Male	626 (90%)	71 (10%)		2.43	1.77–3.35
Female	530 (78%)	150 (22%)		1.0 (ref)	
Age			*P* = 0.072		
16–18 years	769 (85%)	134 (15%)		1.29	0.94–1.77
13–15 years	390 (81%)	89 (19%)		1.0 (ref)	
Residence			*P* < 0.001		
Farm	660 (91%)	67 (9%)		4.19	2.99–5.88
Country/not a farm	216 (86%)	34 (14%)		2.74	1.78–4.24
Town	279 (70%)	121 (30%)		1.0 (ref)	
Race			*P* < 0.001		
White/Caucasian	1,118 (85%)	202 (15%)		1.0 (ref)	
Other races	40 (66%)	21 (34%)		0.43	0.24–0.78

a The actual response was “Not that I know of” as homes may have had firearms but the adolescent respondent may not have known that they were present.

b The sum of n for a variable may not equal the total Group N due to missing values.

**Table 3:** Bivariate and multivariable logistic regression analyses regarding the presence of handguns in the homes of adolescent survey respondents.

	Crosstab analysis			Logistic regression analysis	
	
	Yes n (Row %)^b^	No^a^ n (Row %)^b^	P-value	Odds ratio	Confidence interval
Group N	580 (42%)	802 (58%)			
Gender			*P* = 0.005		
Male	430 (62%)	267 (38%)		1.35	1.08–1.68
Female	369 (54%)	311 (46%)		1.0 (ref)	
Age			*P* = 0.358		
16–18 years	516 (57%)	387 (43%)		0.88	0.77–1.11
13–15 years	286 (60%)	193 (40%)		1.0 (ref)	
Residence			*P* < 0.001		
Farm	458 (63%)	269 (37%)		1.70	1.32–2.18
Country/not a farm	143 (57%)	107 (43%)		1.30	0.95–1.80
Town	198 (50%)	202 (50%)		1.0 (ref)	
Race			*P* = 0.370		
White/Caucasian	769 (58%)	551 (42%)		1.0 (ref)	
Other races	32 (52%)	29 (48%)		0.90	0.53–1.52

a The actual response was “Not that I know of” as homes may have had firearms but the adolescent respondent may not have known that they were present.

b The sum of n for a variable may not equal the total Group N due to missing values.

**Table 4:** Storage of firearms and of handguns in the homes of adolescent survey respondents.

	Rifles/shotguns n (Col %)^a^	Handguns n (Col %)^b^
Stored loaded		
No	731 (69%)	472 (60%)
Yes, sometimes	219 (21%)	170 (21%)
Yes, always	112 (11%)	151 (19%)
Stored unlocked		
No	521 (47%)	400 (54%)
Yes, sometimes	337 (30%)	209 (28%)
Yes, always	251 (23%)	133 (18%)
Stored loaded and unlocked		
No	879 (82%)	539 (73%)
Yes, sometimes	136 (13%)	124 (17%)
Yes, always	58 (5%)	71 (10%)
Overall storage		
Safe storage^c^	360 (33%)	275 (37%)
Unsafe storage^d^	716 (67%)	463 (63%)

a Does not include those who had no rifles/shotguns in the home or were unsure of storage.

b Does not include those who had no handguns in the home or were unsure of storage.

c Firearms always stored unloaded and locked.

d Firearms stored at least sometimes loaded and/or unlocked.

**Table 5:** Bivariate and multivariable logistic regression analyses regarding the storage of rifles/shotguns in the homes of adolescent survey respondents.^a^

	Crosstab analysis			Logistic regression analysis	
	
	Yes^b^ n (Row %)^c^	No n (Row %)^c^	P -value	Odds ratio	Confidence interval
Stored loaded					
Gender			*P* = 0.521		
Male	193 (32%)	411 (68%)		1.08	0.83–1.41
Female	137 (30%)	318 (70%)		1.0 (ref)	
Age			*P* = 0.618		
16–18 years	227 (32%)	490 (68%)		1.07	0.81–1.42
13–15 years	104 (30%)	241 (70%)		1.0 (ref)	
Residence			*P* = 0.607		
Farm	195 (32%)	415 (68%)		1.05	0.76–1.45
Country/not a farm	57 (28%)	145 (72%)		0.87	0.58–1.32
Town	77 (31%)	169 (69%)		1.0 (ref)	
Race			*P* = 0.966		
White/Caucasian	319 (31%)	717 (69%)		1.0 (ref)	
Other races	11 (31%)	24 (69%)		1.05	0.51–2.19
Stored unlocked					
Gender			*P* < 0.001		
Male	366 (60%)	247 (40%)		1.83	1.43–2.33
Female	221 (45%)	272 (55%)		1.0 (ref)	
Age			*P* = 0.014		
16–18 years	411 (56%)	328 (44%)		1.34	1.03–1.73
13–15 years	177 (48%)	193 (52%)		1.0 (ref)	
Residence			*P* < 0.001		
Farm	365 (57%)	271 (43%)		1.83	1.35–2.46
Country/not a farm	109 (51%)	103 (49%)		1.40	0.96–2.03
Town	112 (43%)	146 (57%)		1.0 (ref)	
Race			*P* = 0.030		
White/Caucasian	573 (54%)	496 (46%)		1.0 (ref)	
Other races	14 (36%)	25 (64%)		0.53	0.27–1.05
Stored loaded and unlocked					
Gender			*P* = 0.003		
Male	129 (21%)	480 (79%)		0.88	0.67–1.16
Female	65 (14%)	396 (86%)		1.0 (ref)	
Age			*P* = 0.017		
16–18 years	144 (20%)	574 (80%)		1.25	0.93–1.68
13–15 years	50 (14%)	305 (86%)		1.0 (ref)	
Residence			*P* = 0.063		
Farm	125 (20%)	487 (80%)		1.19	0.85–1.65
Country/not a farm	32 (15%)	175 (85%)		0.80	0.51–1.24
Town	36 (14%)	214 (86%)		1.0 (ref)	
Race			*P* = 0.630		
White/Caucasian	186 (18%)	848 (82%)		1.0 (ref)	
Other races	8 (21%)	30 (79%)		1.13	0.54–2.35

a Those who answered “Unsure” regarding firearm storage were not included in that analysis.

b Includes those who answered “Yes, Always” and “Yes, Sometimes.”

c The sum of n for a variable may not equal the total Group N due to missing values.

**Table 6:** Bivariate and multivariable logistic regression analyses regarding the storage of handguns in the homes of adolescent survey respondents.^a^

	Crosstab analysis			Logistic regression analysis	
	
	Yes^b^ n (Row %)^c^	No n (Row %)^c^	P -value	Odds ratio	Confidence interval
Stored loaded					
Gender			*P* = 0.003		
Male	199 (48%)	218 (52%)		1.56	1.15–2.10
Female	119 (37%)	204 (63%)		1.0 (ref)	
Age			*P* = 0.246		
16–18 years	217 (45%)	268 (55%)		1.10	0.81–1.51
13–15 years	104 (40%)	154 (60%)		1.0 (ref)	
Residence			*P* = 0.607		
Farm	176 (41%)	253 (59%)		0.75	0.53–1.07
Country/not a farm	60 (44%)	76 (56%)		0.83	0.52–1.31
Town	84 (48%)	91 (52%)		1.0 (ref)	
Race			*P* = 0.554		
White/Caucasian	310 (43%)	403 (57%)		1.0 (ref)	
Other races	11 (38%)	18 (62%)		0.80	0.46–1.01
Stored unlocked					
Gender			*P* < 0.001		
Male	218 (52%)	199 (48%)		1.74	1.29–2.36
Female	122 (38%)	200 (62%)		1.0 (ref)	
Age			*P* = 0.046		
16–18 years	236 (49%)	248 (51%)		1.25	0.91–1.71
13–15 years	106 (41%)	153 (59%)		1.0 (ref)	
Residence			*P* < 0.001		
Farm	215 (51%)	201 (49%)		1.58	1.10–2.27
Country/not a farm	54 (39%)	83 (61%)		1.00	0.63–1.60
Town	71 (39%)	109 (61%)		1.0 (ref)	
Race			*P* = 0.070		
White/Caucasian	333 (47%)	378 (53%)		1.0 (ref)	
Other races	9 (30%)	21 (70%)		0.52	0.23–1.16
Stored loaded and unlocked					
Gender			*P* < 0.001		
Male	143 (34%)	274 (66%)		2.65	1.84–3.83
Female	50 (16%)	264 (84%)		1.0 (ref)	
Age			*P* = 0.048		
16–18 years	138 (29%)	339 (71%)		1.27	0.88–1.84
13–15 years	57 (22%)	200 (78%)		1.0 (ref)	
Residence			*P* = 0.063		
Farm	119 (29%)	297 (71%)		1.51	0.76–2.24
Country/not a farm	36 (26%)	103 (74%)		1.31	0.76–2.24
Town	38 (22%)	138 (78%)		1.0 (ref)	
Race			*P* = 0.759		
White/Caucasian	188 (27%)	516 (73%)		1.0 (ref)	
Other races	7 (24%)	22 (76%)		0.95	0.39–2.31

a Those who answered “Unsure” regarding firearm storage were not included in that analysis.

b Includes those who answered “Yes, Always” and “Yes, Sometimes.”

c The sum of n for a variable may not equal the total Group N due to missing values.

**Table 7:** Bivariate and multivariable logistic regression analyses regarding the storage of rifle/shotgun and handgun ammunition in the homes of adolescent survey respondents.^a^

	Crosstab analysis				Logistic regression analysis	
	
	Ammunition not locked n (Row %)^b^	Ammunition locked with firearms n (Row %)^b^	Ammunition locked separately n (Row %)^b^	P -value	Odds ratio	Confidence interval
Rifle/shotgun ammunition storage						
Group N	291 (28%)	319 (31%)	431 (41%)			
Gender				*P* = 0.148		
Male	178 (30%)	171 (28%)	253 (42%)		1.16	0.88–1.54
Female	112 (26%)	147 (34%)	177 (41%)		1.0 (ref)	
Age				*P* = 0.012		
16–18 years	216 (31%)	203 (29%)	283 (40%)		1.59	1.17–2.16
13–15 years	75 (22%)	116 (34%)	148 (44%)		1.0 (ref)	
Residence				*P* = 0.011		
Farm	181 (30%)	172 (29%)	247 (41%)		1.86	1.29–2.69
Country/not a farm	62 (32%)	60 (31%)	72 (37%)		2.03	1.30–3.16
Town	47 (19%)	86 (35%)	111 (45%)		1.0 (ref)	
Race				*P* = 0.299		
White/Caucasian	310 (31%)	413 (41%)	284 (28%)		1.0 (ref)	
Other races	8 (24%)	18 (55%)	7 (21%)		0.74	0.31–1.74
Unlocked rifles c				*P* < 0.001		
Yes	235 (43%)	133 (24%)	179 (33%)		Not in the Analysis	
No	52 (11%)	179 (38%)	241 (51%)			
Handgun ammunition storage						
Group N	176 (25%)	255 (36%)	282 (40%)			
Gender				*P* = 0.030		
Male	115 (28%)	136 (33%)	155 (38%)		1.93	1.38–2.71
Female	60 (20%)	117 (38%)	127 (42%)		1.0 (ref)	
Age				*P* = 0.111		
16–18 years	126 (27%)	158 (34%)	182 (29%)		1.31	0.92–1.86
13–15 years	50 (20%)	97 (39%)	100 (40%)		1.0 (ref)	
Residence				*P* = 0.318		
Farm	107 (26%)	136 (33%)	164 (40%)		1.75	1.17–2.64
Country/not a farm	33 (26%)	51 (40%)	44 (34%)		1.51	0.9–2.51
Town	35 (20%)	67 (38%)	73 (42%)		1.0 (ref)	
Race				*P* = 0.370		
White/Caucasian	245 (36%)	267 (39%)	171 (25%)		1.0 (ref)	
Other races	9 (31%)	15 (52%)	5 (17%)		0.69	0.27–1.78
Unlocked handguns^c^				*P* < 0.001		
Yes	121 (38%)	89 (28%)	110 (34%)		Not in the Analyses	
No	49 (13%)	157 (42%)	168 (45%)			

a Those who answered “Unsure” regarding firearm storage were not included in that analysis.

b The sum of n for a variable may not equal the total Group N due to missing values.

c Firearms stored at least sometimes unlocked.

## References

[1] Centers for Disease Control and Prevention (CDC) (1997). Rates of homicide, suicide, and firearm-related death among children--26 industrialized countries. MMWR Morb Mortal Wkly Rep.

[2] Cunningham RM, Walton MA, Carter PM (2018). The major causes of death in children and adolescents in the United States. N Engl J Med.

[3] Grinshteyn E, Hemenway D (2016). Violent death rates: the US compared with other high-income OECD countries, 2010. Am J Med.

[4] Centers for Disease Control and Prevention (2020). Web-based injury statistics query and reporting system.

[5] Hamilton EC, Miller CC, Cox CS (2018). Variability of child access prevention laws and pediatric firearm injuries. J Trauma Acute Care Surg.

[6] Kalesan B, Vyliparambil MA, Bogue E (2016). Race and ethnicity, neighborhood poverty and pediatric firearm hospitalizations in the United States. Ann Epidemiol.

[7] Fowler KA, Dahlberg LL, Haileyesus T (2017). Childhood firearm injuries in the United States. Pediatrics.

[8] Azrael D, Cohen J, Salhi C (2018). Firearm storage in gun-owning households with children: results of a 2015 national survey. J Urban Health.

[9] Hamilton D, Lemeshow S, Saleska JLK (2018). Who owns guns and how do they keep them? The influence of household characteristics on firearms ownership and storage practices in the United States. Prev Med.

[10] Scott J, Azrael D, Miller M (2018). Firearm storage in homes with children with self-harm risk factors. Pediatrics.

[11] Grossman DC (2018). Reducing youth firearm suicide risk: evidence for opportunities. Pediatrics.

[12] Sullivan EMA, Joseph L, Simon TR (2015). Suicide trends among persons aged 10–24 years - United States, 1994–2012. MMWR Morb Mortal Wkly Rep.

[13] Elnour AA, Harrison J (2008). Lethality of suicide methods. Inj Prev.

[14] Grossman DC, Reay DT, Baker SA (1999). Self-inflicted and unintentional firearm injuries among children and adolescents: the source of the firearm. Arch Pediatr Adolesc Med.

[15] Carr BG, Nance ML, Branas CC (2012). Unintentional firearm death across the urban-rural landscape in the United States. J Trauma Acute Care Surg.

[16] Nance ML, Carr BG, Kallan MJ (2010). Variation in pediatric and adolescent firearm mortality rates in rural and urban US counties. Pediatrics.

[17] APM Research Lab (2019). APM survey: Americans’ views on key gun policies. Part three: mandating that guns be stored with locks in place.

[18] Cunningham RM, Carter PM, Ranney ML (2019). Prevention of firearm injuries among children and adolescents: consensus-driven research agenda from the Firearm Safety Among Children and Teens (FACTS) Consortium. JAMA Pediatr.

[19] Parker K, Horowitz J, Igielnik R (2017). America’s complex relationship with guns.

[20] Gallup Poll (2019). In Depth: Topics A to Z. Guns.

[21] Cook P, Ludwig J (1996). Guns in America: results of a comprehensive national survey on firearms ownership and use.

[22] Sadowski LS, Munoz SR (1996). Nonfatal and fatal firearm injuries in a rural county. JAMA.

[23] Senturia YD, Christoffel KK, Donovan M (1994). Children’s household exposure to guns: a pediatric practice-based survey. Pediatrics.

[24] Shaughnessy AF, Cincotta JA, Adelman A (1999). Family practice patients’ attitudes toward firearm safety as a preventive medicine issue: a HARNET Study. Harrisburg Area Research Network. J Am Board Fam Pract.

[25] Nordstrom DL, Zwerling C, Stromquist AM (2001). Rural population survey of behavioral and demographic risk factors for loaded firearms. Inj Prev.

[26] Dresang LT (2001). Gun deaths in rural and urban settings: recommendations for prevention. J Am Board Fam Pract.

[27] Guetschow B, Lilienthal M, Willey M (2018). Unintentional firearm injuries remain prevalent over a 12 year experience at a rural Midwestern Level 1 trauma center. Iowa Orthop J.

[28] Dodge GG, Cogbill TH, Miller GJ (1994). Gunshot wounds: 10-year experience of a rural, referral trauma center. Am Surg.

[29] Rowhani-Rahbar A, Oesterle S, Skinner ML (2020). Initiation age, cumulative prevalence, and longitudinal patterns of handgun carrying among rural adolescents: a multistate study. J Adolesc Health.

[30] Substance Abuse and Mental Health Services Administration (SAMHSA) (2018). 2017 NSDUH Detailed Tables.

[31] Igielnik R (2017). Rural and urban gun owners have difference experiences, views on gun policy.

[32] US Department of the Interior, US Fish and Wildlife Service, U.S. Department of Commerce et al (2018). 2016 National Survey of Fishing, Hunting, and Wildlife-Associated Recreation.

[33] Yamane D (2017). The sociology of U.S. gun culture. Sociol Compass.

[34] Livingston M, Lee M (1992). Attitudes toward firearms and reasons for firearm ownership among nonurban youth: salience of sex and race. Psychol Rep.

[35] Morgan ER, Gomez A, Rowhani-Rahbar A (2018). Firearm ownership, storage practices, and suicide risk factors in Washington state, 2013–2016. Am J Public Health.

[36] Siegel MB, Boine CC (2020). The meaning of guns to gun owners in the US: the 2019 National Lawful Use of Guns Survey. Am J Prev Med.

[37] Aitken ME, Minster SD, Mullins SH (2020). Parents’ perspectives on safe storage of firearms. J Community Health.

[38] Kellermann AL, Reay DT (1986). Protection or peril? An analysis of firearm-related deaths in the home. N Engl J Med.

[39] Kellermann AL, Rivara FP, Rushforth NB (1993). Gun ownership as a risk factor for homicide in the home. N Engl J Med.

[40] Hemenway D, Solnick SJ (2015). Children and unintentional firearm death. Inj Epidemiol.

[41] Branas CC, Nance ML, Elliott MR (2004). Urban-rural shifts in intentional firearm death: different causes, same results. Am J Public Health.

[42] Herrin BR, Gaither JR, Leventhal JM (2018). Rural versus urban hospitalizations for firearm injuries in children and adolescents. Pediatrics.

[43] Dowd MD, Sege RD (2012). Firearm-related injuries affecting the pediatric population. Pediatrics.

[44] Santaella-Tenorio J, Cerdá M, Villaveces A (2016). What do we know about the association between firearm legislation and firearm-related injuries?. Epidemiol Rev.

[45] Cummings P, Grossman DC, Rivara FP (1997). State gun safe storage laws and child mortality due to firearms. JAMA.

[46] Webster DW, Vernick JS, Zeoli AM (2004). Association between youth-focused firearm laws and youth suicides. JAMA.

[47] Webster DW, Starnes M (2000). Reexamining the association between child access prevention gun laws and unintentional shooting deaths of children. Pediatrics.

[48] Lee J, Moriarty KP, Tashjian DB (2013). Guns and states: pediatric firearm injury. J Trauma Acute Care Surg.

[49] Hepburn L, Azrael D, Miller M (2006). The effect of child access prevention laws on unintentional child firearm fatalities, 1979–2000. J Trauma.

[50] DeSimone J, Markowitz S, Xu J (2013). Child access prevention laws and nonfatal gun injuries. South Econ J.

[51] Azad HA, Monuteaux MC, Rees CA (2020). Child access prevention firearm laws and firearm fatalities among children aged 0 to 14 Years, 1991–2016. JAMA Pediatr.

[52] Evans EM, Jennissen CA, Oral R (2017). Child welfare professionals’ determination of when children’s access or potential access to loaded firearms constitutes child neglect. J Trauma Acute Care Surg.

[53] Jennissen CA, Evans EM, Karsjens AA (2019). Social workers’ determination of when children’s access or potential access to loaded firearms constitutes child neglect. Inj Epidemiol.

[54] Simonetti JA, Rowhani-Rahbar A, King C (2018). Evaluation of a community-based safe firearm and ammunition storage intervention. Inj Prev.

